# Difficulties in Diagnosis and Therapy of Infective Endocarditis in Children and Adolescents—Cohort Study

**DOI:** 10.3390/healthcare9060760

**Published:** 2021-06-19

**Authors:** Alina-Costina Luca, Alexandrina-Stefania Curpan, Heidrun Adumitrachioaiei, Ioana Ciobanu, Cezarina Dragomirescu, Raluca-Stefania Manea, Ecaterina Vlad, Alina Surguci-Copaceanu

**Affiliations:** 1 Sfânta Maria’ Emergency Children’s Hospital, 700309 Iasi, Romania; acluca@yahoo.com (A.-C.L.); ad.heidi91@gmail.com (H.A.); dr.ciobanuioana@gmail.com (I.C.); c.dragomirescu@yahoo.com (C.D.); raluca.mircea@hotmail.com (R.-S.M.); katerynamitioglo@yahoo.com (E.V.); dr.alina.surguci@gmail.com (A.S.-C.); 2 Department of Pediatric Cardiology, Faculty of Medicine, Gr. T. Popa’ University of Medicine and Pharmacy, 700115 Iasi, Romania; 3 Department of Biology, Faculty of Biology, Alexandru Ioan Cuza University, 700505 Iasi, Romania

**Keywords:** infective endocarditis, pathogens, staphylococcus, antibiotics, endocardium

## Abstract

Despite the progress in management and prophylaxis measures, infective endocarditis (IE) is still a condition associated with high mortality rates and severe complications. Fortunately, the incidence of IE is much lower in children and adolescents, with only 0.05–0.12/1000 cases being reported in hospitalized pediatric patients. According to recent data, IE is, in most cases, a complication of pre-existing congenital heart disorders, in up to 75–90% of cases. About 8–10% of all IE cases occur in children without a pre-existing heart condition, due to the widespread use of catheters and invasive procedures, or are associated with immunosuppression. The overall mortality rate due to IE among children and adolescents is 16–25%, a fairly high incidence despite advances made in management and treatment methodologies. We present a retrospective case study conducted in the Pediatric Cardiology Department of ‘St. Maria’ Emergency Children’s Hospital of Iași between February 2007 and February 2020, including 54 children aged between 23 days and 17 years. Our study was aimed at revealing the evolution of IE in recent years in the pediatric population, at identifying the main causes leading to the onset and progress of the disease, at assessing the incidence of clinical and paraclinical manifestations and at determining efficient diagnosis and therapy approaches for the population under survey.

## 1. Introduction

Infective endocarditis (IE) is an inflammatory process consisting of colonization and invasion of the endocardium by a pathogenic microorganism, causing the formation of vegetation [1]. Infection most frequently affects the heart valves (native or prosthetic), but it may also occur in the reduced pressure section of the ventricular septum (site of a defect), in areas of the endocardium damaged by abnormal blood jets or foreign bodies, or on intracardiac devices. The analogous process that affects arteriovenous shunts, arterioarterial shunts (patent arterial duct) or areas of aortic coarctation is called infective endarteritis [2]. 

The clinical picture of IE includes a wide range of symptoms, which are mostly due to: virulence of the etiological microorganism, persistence of bacteremia, extent of tissue damage and hemodynamic consequences of the resulting valvulopathies, perivalvular extension of the infection, septic pulmonary and systemic circulation embolisms and consequences of circulating immune complexes [3]. 

Due to the complexity of this condition and its high morbidity and mortality rates, it is imperative to quickly set the diagnosis, begin effective treatment and recognize possible complications. The diagnosis of IE is based on the presence of positive blood cultures and evidence of the presence of intracardiac vegetation.

Medical imaging methods, mainly echocardiography, play a key role in both the diagnosis and follow-up of patients with IE. The usefulness of echocardiography may be extended, so it has become the method of choice for evaluating the prognosis of IE patients, for their follow-up antibiotic therapy, intra- and postoperatively. There are three major echocardiographic criteria for the positive diagnosis of IE: vegetation, abscess and new dehiscence of a prosthetic valve. However, the new guidelines recommend the inclusion of other medical imaging techniques: multi-slice computed tomography (MSCT), magnetic resonance imaging (MRI) and positron emission computed tomography (PET-CT) with 18F-fluorodeoxyglucose.

Another basic pillar in setting an IE diagnosis is microbiological diagnosis. Blood cultures are positive in about 85% of all IE cases, except for blood culture-negative IE (BCNIE) which is generally caused by prior antibiotic therapy. In these cases, antibiotic therapy must be discontinued, and blood cultures must be repeated. Such cases require serological testing, immunological techniques or molecular biology or histological techniques.

Duke criteria based on clinical, echocardiographic and microbiological data are used in current practice. Despite the usefulness of these criteria, they should not replace clinical judgment. Corroboration with the findings of additional medical imaging techniques, such as MSCT, MRI and PET-CT, is useful in detecting silent vascular phenomena and endocardial lesions, and contribute to improving the sensitivity of Duke criteria. Thus, the ESC Guidelines Committee (2015) suggested the implementation of three new diagnosis criteria: [4]

1. Identification of paravalvular lesions by heart CT (major criterion).
2. In case of suspicion of valve prosthesis EI, detection of abnormal activity at the prosthesis implantation site (only if the prosthesis was implanted more than 3 months before) by 18F-FDG PET/CT or SPECT/CT with radio-traced leukocytes (major criterion).
3. Identification of recent embolic events or infectious aneurysms only by medical imaging techniques (silent events) (minor criterion).

Our study is aimed at revealing the evolution of IE in recent years in the pediatric population, at identifying the main causes leading to the onset and progress of the disease, at assessing the incidence of clinical and paraclinical manifestations and at determining efficient diagnosis and therapy approaches for the population under survey.

## 2. Materials and Methods

### Patient Recruitment and Research Methodology

This is a retrospective case study conducted in the Pediatric Cardiology Department of ‘St. Maria’ Emergency Children’s Hospital of Iași between February 2007 and February 2020, a period in which we evaluated a total of 45,258 pediatric patients, from which we included the 54 children (33 males vs. 21 females) diagnosed with infective endocarditis, giving an incidence of 0.11% in our hospital. The patients were chosen based on the European Cardiology Society guidelines following Duke inclusion criteria. The present study was conducted according to Romanian research law no. 206/27.05.2004 as well as the European laws. The parents and children were informed about the study, what it involved and what information was going to be used, and approval from the Ethics Committee was obtained with the registration number 14,355/14.05.2021. The patients’ ages varied from 23 days to 17 years old and we observed the highest incidence in the 11–17-year-old age group (19 cases) with a higher prevalence in females (33%) and in children from rural areas, as shown in Table 1.

We analyzed the epidemiological data, the main clinical and paraclinical manifestations, their incidence in the surveyed pediatric population, the main complications and therapeutic approaches in order to reach the final results and draw the final conclusions.

## 3. Results

**Figure 1:**
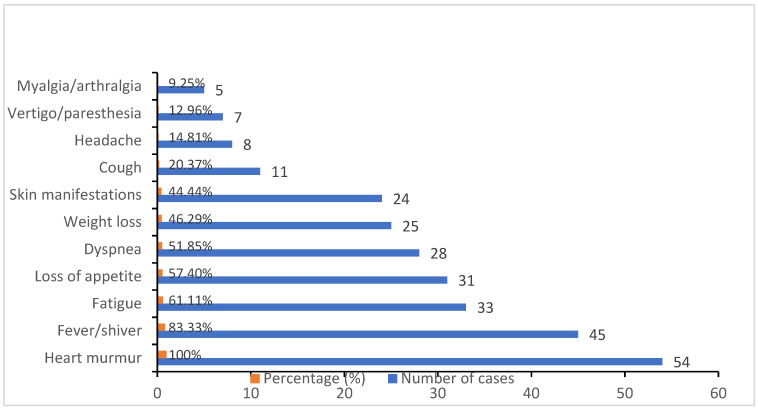
Prevalence of clinical manifestations at the time of hospital admission illustrated by percentages and the corresponding number of cases.

In the case of our study, we had a higher prevalence of male patients (33 males vs. 21 females) and of patients from rural areas (32 rural vs. 22 urban). The clinical picture of patients during their hospitalization was: heart murmurs (100%), followed by fever (83.33%), fatigue (61.11%), reported loss of appetite (57.40%) and dyspnea (51.85%). Other clinical manifestations presented were weight loss (46.29%) and skin manifestations such as petechiae, Janeway lesions and Roth spots (44.44%), followed by cough (20.37%), headache (14.81%), vertigo (12.96%) and myalgias (9.25%) to a lesser extent, as shown in Figure 1.

**Figure 2:**
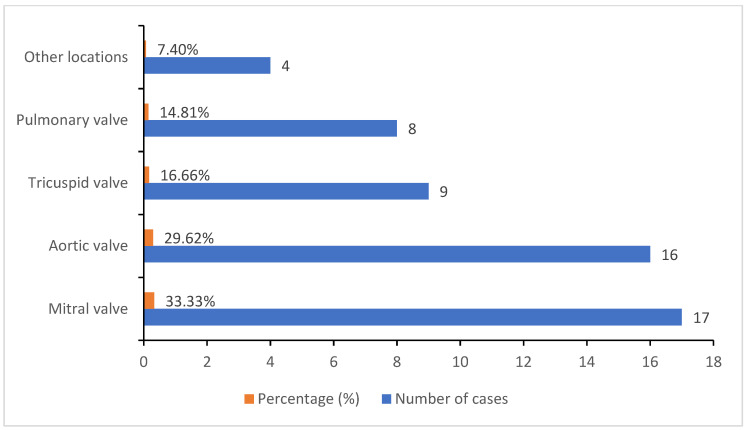
Echocardiographic distribution of valve injuries illustrated by percentages and the corresponding number of cases.

In about 85% (46 cases) of our patients, infective endocarditis was secondary to a pre-existing heart condition, especially valve damage (mainly mitral and aortic valve injury) and congenital heart malformations (more commonly ventricular septal defect, atrial septal defect, patent ductus arteriosus, coarctation of the aorta). All of our patients were examined by echocardiography, which revealed major criteria for diagnosis of infective endocarditis. The dimensions of the vegetations identified through echocardiography varied and can be observed in Table 2. In terms of injury localization, the mitral valve (33.33%) and the aortic valve (29.62%) were most commonly impaired, followed by the tricuspid valve (16.66%) and the pulmonary valve (14.81%) (Figure 2).

**Figure 3:**
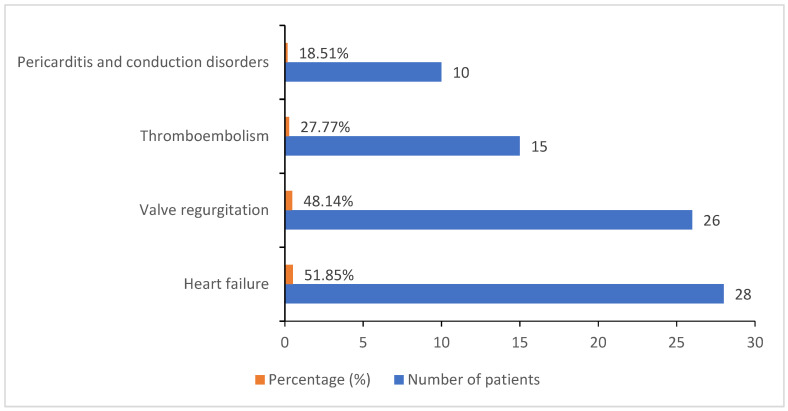
Infective endocarditis complications illustrated in percentages and the corresponding number of cases.

Besides the abovementioned clinical manifestations, we also observed a series of several complications during the progression of infective endocarditis, the most common of which were heart failure (51.85%) and valve regurgitation (48.14%), followed by thromboembolic conditions (27.77%), and, with a lower incidence, pericarditis and conduction disorders, which occurred in 10 of the total complicated cases (18.51%) (Figure 3).

We used blood samples to obtain blood cultures. Blood samples were acquired using BD BACTEC Peds plus/F vials. For newborns, nurslings and children up to 2 years old, 1–3 mL of blood is recommended to be collected, and after this age the amount of collected blood increases dependent on the age of the patient. After blood collection, the vials were placed in BD BACTEC FX40 and incubated at 35 ± 1 °C for 5 days. From the positive vials, we Gram-stained smears and examined them with a microscope with immersion. Based on the microscope results, we sub-cultivated on adequate solid mediums: Columbia agar with sheep blood, chocolate blood agar, MacConkey agar and Sabouraud agar. We carried out the angiogram and the biochemical identification of the strain using a MicroScan WalkAway 40 Plus System. The most common pathogen found was *Staphylococcus aureus*, followed by *Viridans Streptococci* and the HACEK group. The blood cultures were negative in nine patients, those that were on antibacterial treatment prior to admission. The therapeutic approach considered the antibiotic sensitivity of the identified microorganisms. For treatment, we used different combinations of antibiotics from the cephalosporin 3rd generation, aminoglycoside, carbapenem and glycopeptide classes. Therefore, the blood cultures became negative during the first week of antibacterial treatment in most patients. However, the therapy was continued until the intracardiac vegetation became sterile according to international guidelines. Sterilization was deemed successful in all patients. Only one patient required surgical therapy, with a positive post-surgical evolution.

Patients were also reexamined by cardiological and echocardiographic means to assess therapy effectiveness and dynamic heart function. Due to their early diagnosis and prompt therapy, the evolution of the 48 patients included in the study (88.88%) was positive. Infective endocarditis was fatal in six of the patients (11.11%), as they all had severe pre-existing heart conditions and one patient was diagnosed with terminal phase oncologic pathology.

## 4. Discussion

The clinical picture of IE includes a wide range of symptoms. A new heart murmur is common, especially in patients with IE complicated by heart failure. Splenomegaly is found in about 50% of patients. Neurological complications, such as embolic stroke, brain abscesses, fungal aneurysms and intracerebral hemorrhages, are late manifestations and are usually associated with staphylococcal infections. Myocardial abscesses are generally specific to staphylococcal infections and may cause conduction disorders, blockages or, when the injury occurs in the pericardium, purulent pericarditis. A number of classical peripheral signs are described, which are generally late manifestations: petechiae (most common in the conjunctiva, oral mucosa and extremities), Janeway lesions (erythematous or hemorrhagic maculae located on the palms or soles, sequelae of peripheral septic embolism associated *Staphylococcus aureus* IE), subungual hemorrhages, Osler nodules (painful, firm subcutaneous nodules in the fingers, the result of deposits of immune complexes) and Roth spots (retinal hemorrhage detected in 10–20% of cases) [5], some of which were also identified in our patients.

*Viridans*-type streptococci and *Staphylococcus aureus* remain the leading causative agents for endocarditis in pediatric patients [5]. Fungal endocarditis is rare both in children and adults. Although injected drug use was traditionally an important risk factor for *Candida* IE, care contact has now emerged as the primary risk factor for most patients with this infection. The overall cure rate in cases of fungal IE is poor [6]. The poor prognosis may be due to large, bulky vegetations; a tendency for fungal invasion of the myocardium; widespread systemic septic emboli; poor penetration of antifungal agents into the vegetation; low toxic-to-therapeutic ratio of the available antifungal agents and the usual lack of fungicidal activity with these compounds [6]. A cure is almost impossible without surgical intervention.

The 2015 European Guide to Infective Endocarditis Management highlights the major role of a multidisciplinary approach, involving a pediatric cardiologist, cardiovascular surgeon, infectious disease specialist, microbiologist and medical imaging specialist. The IE therapy principles are microbial eradication through the use of antibiotics; removal of infected material; drainage of abscesses and repair of valve damage/valve prosthesis [4]. Extended antimicrobial therapy is the basis of IE treatment, and the treatment of IE on valve prostheses should last longer (at least 6 weeks), while the treatment of IE on native valves should last 2–6 weeks. In both cases, treatment duration is determined by the first day on which antibiotic therapy is effective and not by the day of surgery. A new treatment plan should be started only if the valve cultures are positive, with the choice of antibiotic being based on the susceptibility of the last isolated bacteria [4]. In some cases, antibacterial therapy should be supplemented with surgical treatment to eradicate the infection focus. The clinical signs that mark the necessity of surgery are congestive heart failure secondary to valvular dysfunction or prosthetic material dehiscence; growing vegetation and its embolism; perivalvular extension of myocardial abscess and complicated myocardial abscess with atrioventricular block.

A retrospective case study conducted in the USA between 2000 and 2010 on a group of 3840 children diagnosed with IE, with a pediatric population incidence of 0.43/100,000, more than half of whom were aged over 11 years, revealed that 30% of the cases had negative blood cultures. *Staphylococcus* species were the most common pathogen in the positive cultures, followed by *Streptococcus* species [7]. In the case of our study, only 16.66% of the total patients had negative blood cultures, but the most common pathogen was indeed *Staphylococcus* followed by a *Streptococcus.*

Another large-scale study conducted in Norway at the Clinical Teaching Hospital for Congenital Heart Malformations between 1994 and 2016 showed a much higher incidence among patients with congenital heart disease, i.e., 2.2/10,000/year. Most of these patients (75%) had severe congenital heart diseases (CHDs) and had undergone open heart surgery during the last year prior to IE diagnosis setting [8].

In a retrospective study conducted in the USA between 2003 and 2014 in 29 centers on 841 children diagnosed with IE, Bates et al. studied the impact of the antibiotic prophylaxis guidelines suggested by the AHA in 2007. The study concluded that there was no significant decrease in the hospitalization rate of IE children after 2007, with the infection rate decreasing from 0.13 cases/10,000 hospitalizations for 6 months to 0.12 cases/10,000 for 6 months [9].

Life-threatening complications include stroke and intracerebral and subarachnoid hemorrhage. Thus, in a 13-year study conducted in China between January 2002 and December 2015 on 60 children with IE, the neurology department of Beijing Anzhen Hospital found a higher incidence of stroke in children with IE of the left half of the heart than in those with IE in the right half of the heart (32% compared to 2.8%). The most common manifestation of stroke was hemiparesis (55.5%), and the mortality rate was significantly higher in patients with stroke than in those who did not suffer any stroke (22.2% compared to 3.9%) [10].

The Italian Society for Pediatric Infectious Diseases conducted a retrospective study on 47 IE patients aged 2 to 17 years between 2000 and 2015 and concluded that the most common pathogen was *Streptococcus viridians* in patients with pre-existing heart disease, and *Staphylococcus aureus* in those without pre-existing heart disease (37.9% compared to 5.5% and 6.9% compared to 27.8%), while 85.7% were methicillin-resistant *S. aureus* [11].

Dixon and Chistov concluded that despite all the advances in its management, pediatric infective endocarditis still poses clinical problems. Improved and more sophisticated laboratory techniques, wider use of echocardiography and the use of new medical imaging methods allow for better diagnosis. However, the increasing number of intracardiac implants (the incidence of pulmonary valves of the bovine jugular vein implanted with a transcatheter is relatively high) leads to a change in predisposing factors and brings about new diagnosis and treatment challenges. The impact of changes in prophylaxis guidelines remains uncertain and there is still no evidence to support their effectiveness [12]. 

A retrospective study conducted at the Rawalpindi Institute of Cardiology between 1 January and 15 October 2017 on 120 pediatric patients with suspicions of IE showed that the incidence in male patients was higher than that in female patients (70% compared to 30%), while the mean age of the patients was 5.5 (± 1.7) years. Additionally, the most common complaints of patients were fever, shortness of breath, chest discomfort and cyanotic episodes. The most common underlying disease associated with endocarditis was congenital heart defect (CHD) (52%, followed by rheumatic heart disease in 32%). Blood cultures were positive in 32% of the patients, while in 68% of them blood cultures were negative. Coagulase-negative *Staphylococcus* was the most common isolated organism (31.2%) in the research patients, followed by *Streptococcus viridians* [13]. Our findings are in agreement with these observations, except the number of positive blood cultures (45 in our case) and the most common pathogen being coagulase-positive *S. aureus*.

Another retrospective study conducted in Belgium at the Department of Pediatric Cardiology, KU Leuven, between 2000 and 2017, showed that the incidence of patients who needed heart surgery was 36%, with the mortality rate being 13% and 87% of the patients with congenital heart defects. The pathogenic factor was detected in 92% of the cases: *Streptococcus viridians* (32%), *S. aureus* (25%), coagulase-negative *Staphylococci* (20%) [14].

As far as medication is concerned, in 2016, Nichols et al. studied the recommendations of the updated IE guidelines published by the AHA in 2015 regarding certain antibiotics, such as: vancomycin, gentamicin and tobramycin, cefepime, piperacillin/tazobactam, nafcillin and penicillin G. Thus, they concluded that the doses listed here followed the vancomycin, aminoglycoside and β-lactam dosing and follow-up recommendations, and they were based primarily on experts’ opinions and failed to take into account the available dose-optimization evidence based on pharmacokinetic and pharmacodynamic principles in children and adolescents. These findings are disconcerting, since, in a clinical setting, some practitioners may be reluctant to deviate from the doses recommended by the guidelines [15].

Despite the positive results of our cases, our study is limited by a small sample size (54 cases), and imbalance between the numbers of each gender, but we believe that our wider age distribution (with the highest prevalence of IE in the 11–17-year-old patients) provides a good insight regarding treatment efficacy in different age groups and different backgrounds. However, the retrospective nature of our paper brings several limitations to our study, as this type of work is more prone to biases and missing information since the cases were first registered for clinical purposes and not research.

## 5. Conclusions

Infective endocarditis is a severe condition that unfortunately also affects pediatric patients, in some cases even being fatal. The presence of pre-existing heart conditions such as congenital heart defects and valvular injuries is a major predisposing factor to infective endocarditis in children.

Medical imaging diagnosis and blood cultures are particularly important for fast and efficient intervention, for successful therapy and for a positive outcome. Our study illustrated the importance of early diagnosis and therapy, with a successful recovery rate of 88.88% in our case, with only one patient needing surgery, and the most common causes were *S. aureus, S. viridans* and the HACEK group for IE. The lethality cause was severe congenital heart defects, which were observed in six of our initial patients. Future studies should focus more on establishing the optimum dosages for the antibacterial therapy as well as developing more personalized therapies based on the observed symptoms and complications of every patient in particular, as it might increase the chance of a favorable outcome.

## Figures and Tables

**Table 1:** Baseline characteristics of the overall cohort.

Characteristics	Infective Endocarditis Patients/Total Pediatric Patients	Proportion
**Diagnosed with infective endocarditis**	54/45,258	0.11% of all cases
Age groups		
Infant (0–1 years)	8	14.8%
Toddler (1–5 years)	12	22.2%
Middle childhood (6–10 years)	15	27.7%
Adolescent (≥11 years)	19	35.1%
Gender groups		
Male	21	38.8%
Female	33	61.1%
Living area		
Rural area	32	59.25%
Urban area	22	40.74%
Pre-existing heart condition	46	85%
Clinical features during hospitalization		
Heart murmurs	54	100%
Fever	45	83.33%
Fatigue	33	61.11%
Loss of appetite	31	57.40%
Dyspnea	28	51.85%
Weight loss	25	46.29%
Skin manifestations (petechiae, Janeway lesions, Roth spots)	24	44.44%
Cough	11	20.37%
Headache	8	14.81%
Vertigo	7	12.96%
Myalgias	5	9.25%
Complications		
Heart failure	28	51.85%
Valve regurgitation	26	48.14%
Thromboembolic conditions	15	27.77%
Pericarditis and conduction disorders	10	18.51%
Negative blood cultures due to prior antibacterial treatment	9	16.66%
Positive evolution	48	88.88%

**Table 2:** The dimensions of the identified vegetations in association with incidence, both in number and percentage, in our studied cases.

Dimension (cm)	Number of Cases	Percentage (%)
1–4.9	32	58.82
5–9.9	16	29.41
>10	6	11.76

## Data Availability

All data is available within the article.
